# Molecular Associations and Clinical Significance of RAPs in Hepatocellular Carcinoma

**DOI:** 10.3389/fmolb.2021.677979

**Published:** 2021-06-21

**Authors:** Sarita Kumari, Mohit Arora, Jay Singh, Lokesh K. Kadian, Rajni Yadav, Shyam S. Chauhan, Anita Chopra

**Affiliations:** ^1^ Laboratory Oncology Unit, Dr. BRA-IRCH, All India Institute of Medical Sciences, New Delhi, India; ^2^ Department of Biochemistry, All India Institute of Medical Sciences, New Delhi, India; ^3^ Department of Pathology, All India Institute of Medical Sciences, New Delhi, India

**Keywords:** hepatocellular carcinoma, liver, RAP, TCGA, prognosis, biomarker 3

## Abstract

Hepatocellular carcinoma (HCC) is an aggressive gastrointestinal malignancy with a high rate of mortality. Multiple studies have individually recognized members of RAP gene family as critical regulators of tumor progression in several cancers, including hepatocellular carcinoma. These studies suffer numerous limitations including a small sample size and lack of analysis of various clinicopathological and molecular features. In the current study, we utilized authoritative multi-omics databases to determine the association of RAP gene family expression and detailed molecular and clinicopathological features in hepatocellular carcinoma (HCC). All five RAP genes were observed to harbor dysregulated expression in HCC compared to normal liver tissues. RAP2A exhibited strongest ability to differentiate tumors from the normal tissues. RAP2A expression was associated with progressive tumor grade, *TP53* and *CTNNB1* mutation status. Additionally, RAP2A expression was associated with the alteration of its copy numbers and DNA methylation. RAP2A also emerged as an independent marker for patient prognosis. Further, pathway analysis revealed that RAP2A expression is correlated with tumor-infiltrating immune cell composition and oncogenic molecular pathways, such as cell cycle and cellular metabolism.

## Introduction

Hepatocellular carcinoma (HCC) is the sixth leading cancer in incidence and the fourth most common cause of cancer mortality in the world (Bray et al., 2018). It is the most common type of primary liver cancer that usually arises on the background of chronic liver disease, hepatitis B or C virus infection, or nonalcoholic steatohepatitis (Bruix et al., 2011; Villanueva, 2019). For locally advanced cancers without cirrhosis, the 5-years survival rate of patients is only 36–70% and 60–70% with successful surgical resection or liver transplantation, respectively. Further, postoperative recurrence and metastasis are common in HCC, which pose a challenge in the management of this disease. Therefore, biomarkers to predict prognosis in HCC are highly needed. The common indicators of prognosis of HCC include tumor size, degree of cirrhosis, tumor differentiation and microvascular invasion (Villanueva, 2019). The recent emergence of high throughput sequencing data by multiple studies has enabled researchers to describe molecular features of HCC in detail and has provided several potential biomarkers for the prediction of patient prognosis (Wheeler and Roberts, 2017).

RAP proteins (Ras proximate proteins) are members of the Ras GTP binding family sharing 50–60% sequence homology with the Ras family. The diversity and specificity of Ras and RAP proteins are contributed by different sets of GEFs (guanine nucleotide exchange factors) and GAPs (GTPase-activating proteins). Five different genes of the RAP family, RAP1A, RAP1B, RAP2A, RAP2B, and RAP2C have been identified in the human genome (Bokoch, 1993). RAP proteins primarily function in cell adhesion, migration, and polarity (Bokoch, 1993; Ehrhardt et al., 2002; Di et al., 2015b; Qu et al., 2016; Meng et al., 2018). The effect of RAP activation depends on the context-specific interaction of RAP with their regulators and downstream effectors.

Oncogenic functions of RAP proteins have been well established in multiple cancer types, such as breast (Di et al., 2015a), lung (Fu et al., 2009; Wu et al., 2014; Xie et al., 2015; Peng et al., 2016), ovary (Che et al., 2015; Lu et al., 2016), stomach (Zhang J. et al., 2020), cervix (Li et al., 2018), prostate (Bigler et al., 2007) and brain (Wang et al., 2017). Accumulating evidence suggests that RAP proteins also play critical roles in hepatocellular carcinogenesis and tumor progression. Single nucleotide polymorphism (SNPs) in RAP1A gene rs494453 has been shown to associate with higher incidence and recurrence after liver transplantation (Mo et al., 2018; Zhang R. et al., 2020). Further, higher activity of the NF-κB/RAP1 signaling pathway is associated with tumorigenicity in HCC cells (Mo et al., 2018). Some studies have also provided strong links between RAP1A expression and liver inflammation, a risk factor for liver carcinogenesis. RAPGEF1, the GEF for RAP1A has also been shown to be overexpressed in HCC (Sequera et al., 2018). A previous study reported that HBV replication promotes liver carcinogenesis through upregulation of RAP1B (Sheng et al., 2014). Further, overexpression of RAP1B enhances the proliferation and migration of HCC cells by regulating Twist-1 gene expression (Tang et al., 2018). Overexpression of RAB2B has also been reported in HCC and its inhibition reduces cell proliferation and invasion (Zhang et al., 2017). Recently, Zheng et al. reported that HCC tissues exhibit significantly higher mRNA and protein expression of RAP2A, which is associated with tumor size, metastasis, pathological differentiation, and vascular invasion (Zheng et al., 2017). Furthermore, they also demonstrated that higher protein levels of RAP2A are independently associated with poor overall survival in HCC.

While the current literature suggests that RAP genes might play critical roles in the pathophysiology of HCC, these studies are limited by determining individual genes of the RAP signaling pathway, limited number of clinical samples used in different studies. Further, studies focused on determining the association of RAP genes with genetic alteration and molecular alterations remain limited. In the current study, we utilized authoritative multi-omics databases to determine the association of RAP gene family expression and detailed molecular and clinicopathological features. Furthermore, we also determined their association with multiple survival parameters to determine their prognostic value.

## Materials and Methods

### Data Retrieval

For mRNA expression analysis, RNA seq data of TCGA-LIHC dataset, which was originally sourced from Broad GDAC Firehose (http://gdac.broadinstitute.org/) (Wheeler and Roberts, 2017) was extracted using UCSC XENA webserver (Goldman et al., 2020). Clinicopathological and molecular characterstics of the TCGA-LIHC dataset has been given in Table 1. Microarray gene expression data from multiple studies was accessed through the TNMplot webserver (Bartha and Győrffy, 2020). This web server hosts data from multiple HCC studies, where gene expression data has been normalized for all available studies and can be used for comparison between the collective groups of all normal samples with tumor samples. Multi-Omics dataset of hepatocellular carcinoma released by Clinical Proteomic Tumor Analysis Consortium (CPTAC) (https://cptac-data-portal.georgetown.edu/cptacPublic/) was utilized to analyze both mRNA and protein levels of RAP genes.

**TABLE 1:** Patient charcterstics in TCGA-LIHC dataset.

Characterstics		Total (370)	%
Age (years)	≤50	75	20.67
	>50	288	79.33
Gender	Male	245	67.30
	Female	119	32.7
Stage	I + II	253	73.76
	III + IV	90	26.24
Grade	I + II	227	63.23
	III + IV	132	36.77
AFP levels	⩽ 400	212	76.81
	> 400	64	23.19
History of alcohol consumption	No	233	66.57
	Yes	117	33.43
Postoperative ablation embolization	No	317	91.88
	Yes	28	8.12
Radiation therapy	No	336	97.67
	Yes	8	2.33
TP53 mutation	No	252	70.19
	Yes	107	29.81
CTNNB1 mutation	No	266	74.09
	Yes	93	25.91
PCLO mutation	No	320	89.13
	Yes	39	10.87
ALB mutation	No	315	87.74
	Yes	44	12.26

### DNA Methylation Analysis

DNA methylation of RAP genes in TCGA cancer dataset was estimated and visualized using MEXPRESS web server (https://mexpress.be) (Koch et al., 2015; Koch et al., 2019) and TCGA Wanderer (Díez-Villanueva et al., 2015). The MEXPRESS web server uses DNA methylation data of cancer and normal tissues from TCGA datasets, which were originally developed on the Illumina Human Methylation 450 BeadChip platform. The predesignated methylation probes for each gene were taken into consideration.

### Survival Analysis

Kaplan-Meier survival analysis was performed using the tool available in the KM-plotter (Nagy et al., 2018). For Kaplan-Meier analysis, patients were distributed in high and low expression groups based on median expression value as a cut-off point for each gene. For survival analysis using univariate and multivariate Cox proportionate hazard model, RAP2A gene expression was taken as a continuous variable with multiple survival parameters for the TCGA-LIHC dataset, as recommended (Liu et al., 2018).

### Correlation and Pathway Enrichment Analysis

Similarly, whole transcriptome correlations of RAP2A in the TCGA-LIHC study were downloaded from the cBioPortal website (https://www.cbioportal.org/) (Cerami et al., 2012; Gao et al., 2013). After applying a filter for a cutoff of FDR corrected p-value of 0.05 for Spearman’s r-value, 10,980 genes with Spearman’s correlation q value <0.05 were filtered and used for gene set enrichment analysis in GSEA software (Broad Institute, http://www.broad.mit.edu/gsea/). Hallmark gene set (version 7.1) (Subramanian et al., 2005) from predefined molecular signature database was used as a reference gene set for pathway enrichment (Liberzon et al., 2015).

### Tumor Immunity Associations

Tumor immune estimation score (TIMER) webserver (https://cistrome.shinyapps.io/timer/), which utilizes the RNA sequencing data from TCGA for estimation of correlation between gene expression and level of immune cells, present in the tumor samples (Li et al., 2017). We utilized TIMER to calculate the association between RAP2A gene expression with infiltration of six immune cells including B cells, CD4+ T cells, CD8+ T cells, neutrophils, macrophages, and dendritic cells in TCGA-LIHC datasets. Default parameters were used in the TIMER database for the gene-specific analysis module.

Further, CIBERSORT (Cell-type Identification By Estimating Relative Subsets Of RNA Transcripts) analysis data of TCGA-LIHC were extracted from a previously published study (Chen et al., 2018; Thorsson et al., 2018). This provided relative fractions of 22 different immune cells from a mixture of gene expression profiles (TCGA-LIHC study) and was used to correlate with RAP2A expression using Spearman’s correlation test. A total of 360 HCC samples were available with both gene expression data and CIBERSORT analysis estimated fractions of immune cells. Heatmap of the immune cell profiling data was generated along with hierarchical clustering using HemI (Deng et al., 2014). The default parameters of hierarchical clustering using the average linkage method and Pearson distance were used.

### Statistical Analysis

Data analysis was performed using Graphpad (version 6) and Stata software (version 11). Mann-Whitney U-test was used for comparison among histological subtypes, molecular subtype and grades (***p < 0.001; **p < 0.01; *p < 0.05; ns, *p* > 0.05). Pearson correlation analysis was used to determine the association of DNA methylation of RAP2A to its expression in the TCGA-LIHC dataset. Kaplan-Meier survival analysis was performed using the log-rank test. A *p*-value < 0.05 was considered statistically significant.

## Results

### Expression Pattern of RAPs in HCC

**FIGURE 1:**
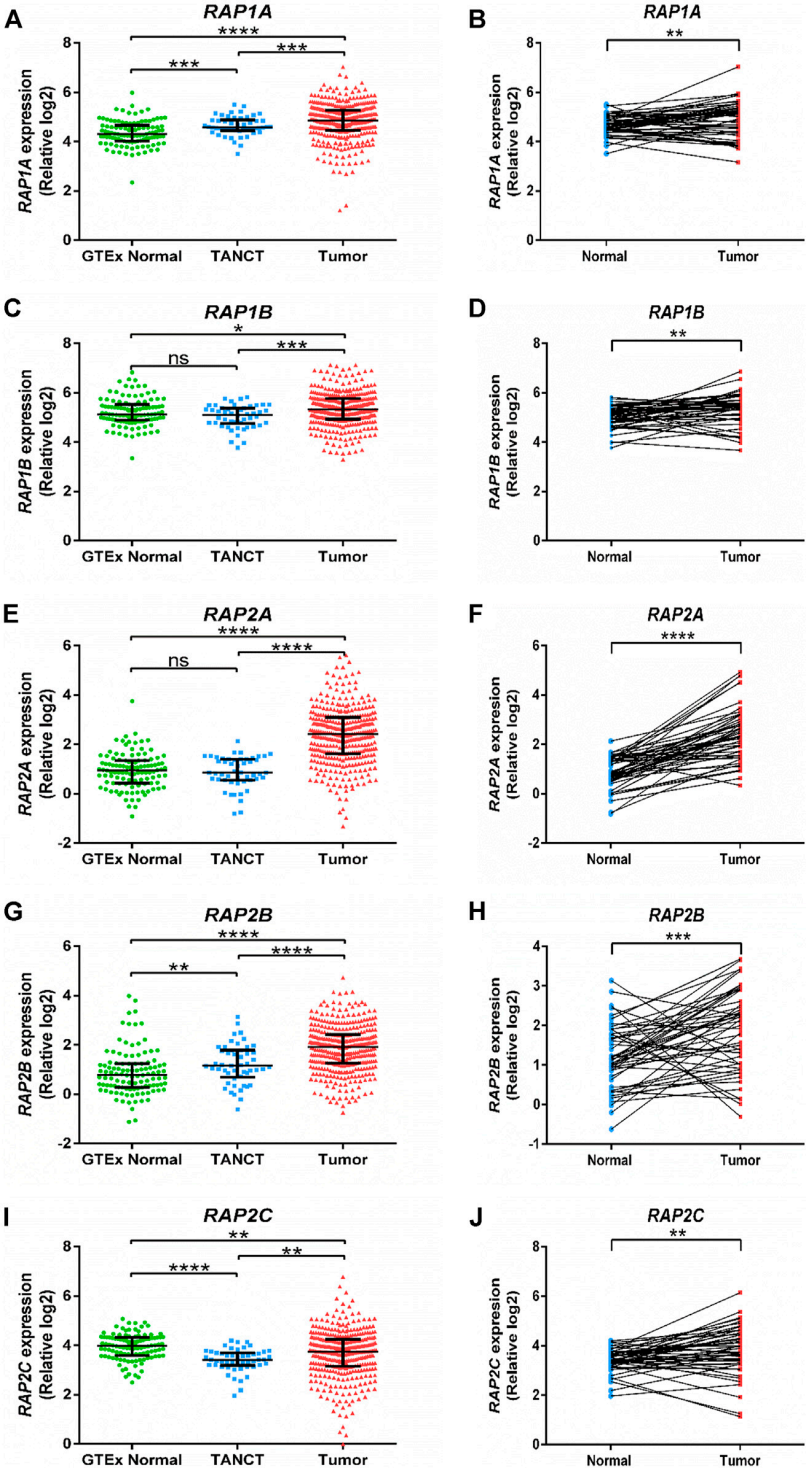
Expression of RAP genes in tumor tissues compared with adjacent normal tissues and normal tissues from GTEx study. GTEx, Genotype-Tissue Expression project; TANCT, tumor adjacent non-cancerous tissue. ****p < 0.0001; ***p < 0.001; **p < 0.01; *p < 0.05; ns, *p* > 0.05.

Further, RNA sequencing data from TCGA-LIHC study was utilized to compare RAP gene expression in tumor tissues with both tumor adjacent normal tissues from the same dataset and with non-tumor associated normal hepatic tissue. RAP1A, RAP1B, RAP2A and RAP2B exhibited significant higher expression in tumors compared to other two groups (Figures 1A,C,E,G). Although RAP2C expression was higher in tumors compared to adjacent normal tissues, but both these groups exhibited lower expression of RAP2C compared to normal tissues from GTEx (Figure 1I). Furthermore, tumor adjacent normal tissues also exhibited higher expression of RAP1A and RAP2B, while no difference was observed for RAP1B and RAP2A. Comparison between 50 paired normal and tumor tissues from TCGA-LIHC also revealed that all RAP genes exhibit higher expression in tumor tissues compared to tumor adjacent normal tissues (Figures 1B,D,F,H,J). Among all RAPs, RAP2A displayed most robust upregulation in tumor tissues in TCGA dataset (Figure 1F). Further, we utilized multiomics data of hepatocellular carcinoma developed by CPTAC study, where both mRNA and proteomic data was available. The expression analysis in CPTAC data also suggested that expression of RAP genes differ between normal and tumor tissues both at the mRNA and protein level. In CPTAC data also, RAP2A exhibited most robust upregulation of mRNA and protein levels in tumor tissues compared to normal tissues, while expression of RAP2C was found to be reduced in tumor tissues compared to normal tissues (Supplementary Figure S1).

**FIGURE 2:**
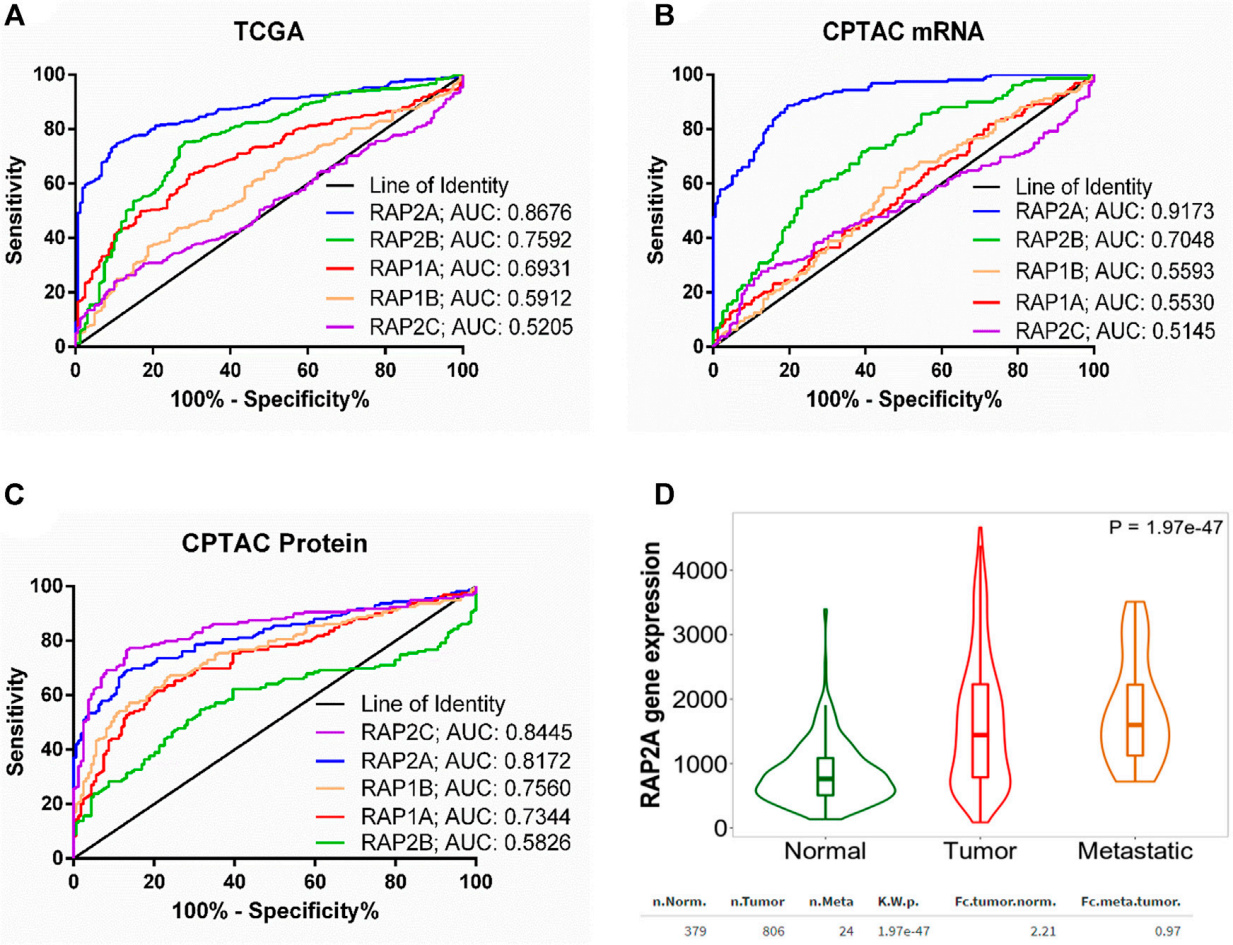
**(A)** ROC curve for the utility of RAP gene expression to differentiate between liver tumor tissues and normal tissue group in **(A)** TCGA mRNA data **(B)** CPTAC mRNA data, and **(C)** CPTAC protein expression data. For **(A)**, normal tissue group consisted of tumor-adjacent normal tissues from TCGA study and normal tissues from non-disease controls from the GTEx study. **(D)** Comparison of RAP2A gene expression among normal tissue, tumor tissue, and metastatic tissues assessed through TNM webtool.

We further performed receiver operating characteristic (ROC) curve analysis to determine potential of RAP gene expression in differentiating tumor tissues from normal liver tissues. Interestingly, among five RAP genes, RAP2A exhibited highest area under curve (AUC) of 0.8676 in TCGA-LIHC mRNA data (Figure 2A). Similarly, analysis of CPTAC mRNA data also suggested highest AUC of RAP2A (AUC: 0.9173, Figure 2B) compared to other RAP genes. Interestingly, analysis of AUC in CPTAC protein expression data revealed that RAP2C exhibit highest AUC of 0.8445 followed by RAP2A with AUC of 0.8172 (Figure 2C).

Furthermore, expression data of RAP gene family in hepatocellular carcinoma tissues and normal tissues from multiple other datasets was assessed through TNMplot web server. The analysis revealed that RAP1A, RAP1B, RAP2A, and RAP2B genes exhibit significantly higher expression in HCC tissues compared to normal tissues in comparison of both available paired (Supplementary Figure S2, left panel) and unpaired tissues (Supplementary Figure S2, right panel). However, RAP2C did not exhibit significant difference in expression in paired tissue analysis (Supplementary Figures S2J). Considering robust upregulation of RAP2A in tumors, and its established involvement in cell migration, we compared expression of RAP2A in metastatic tissues with both normal and primary tissues, which revealed highest expression of RAP2A in metastatic tissues compared to other two groups (Figure 2D).

### Association of RAP Family Expression and Clinicopathological Features in HCC

**FIGURE 3:**
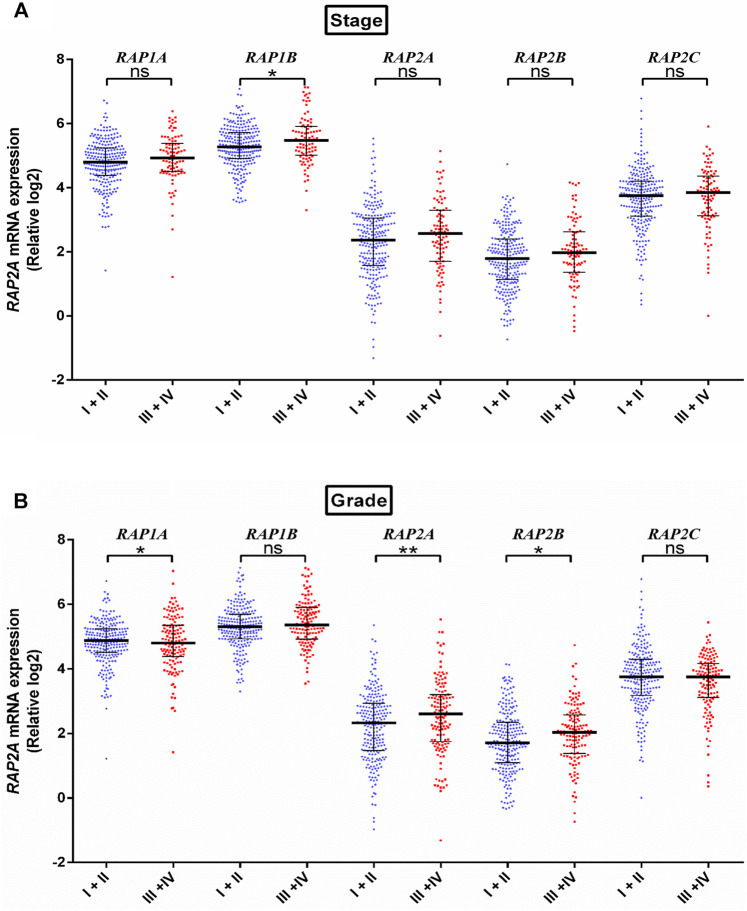
Expression of RAP genes in tumor tissues compared between different stage **(A)** and grade **(B)**. ****p < 0.0001; ***p < 0.001; **p < 0.01; *p < 0.05; ns, *p* > 0.05.

**FIGURE 4:**
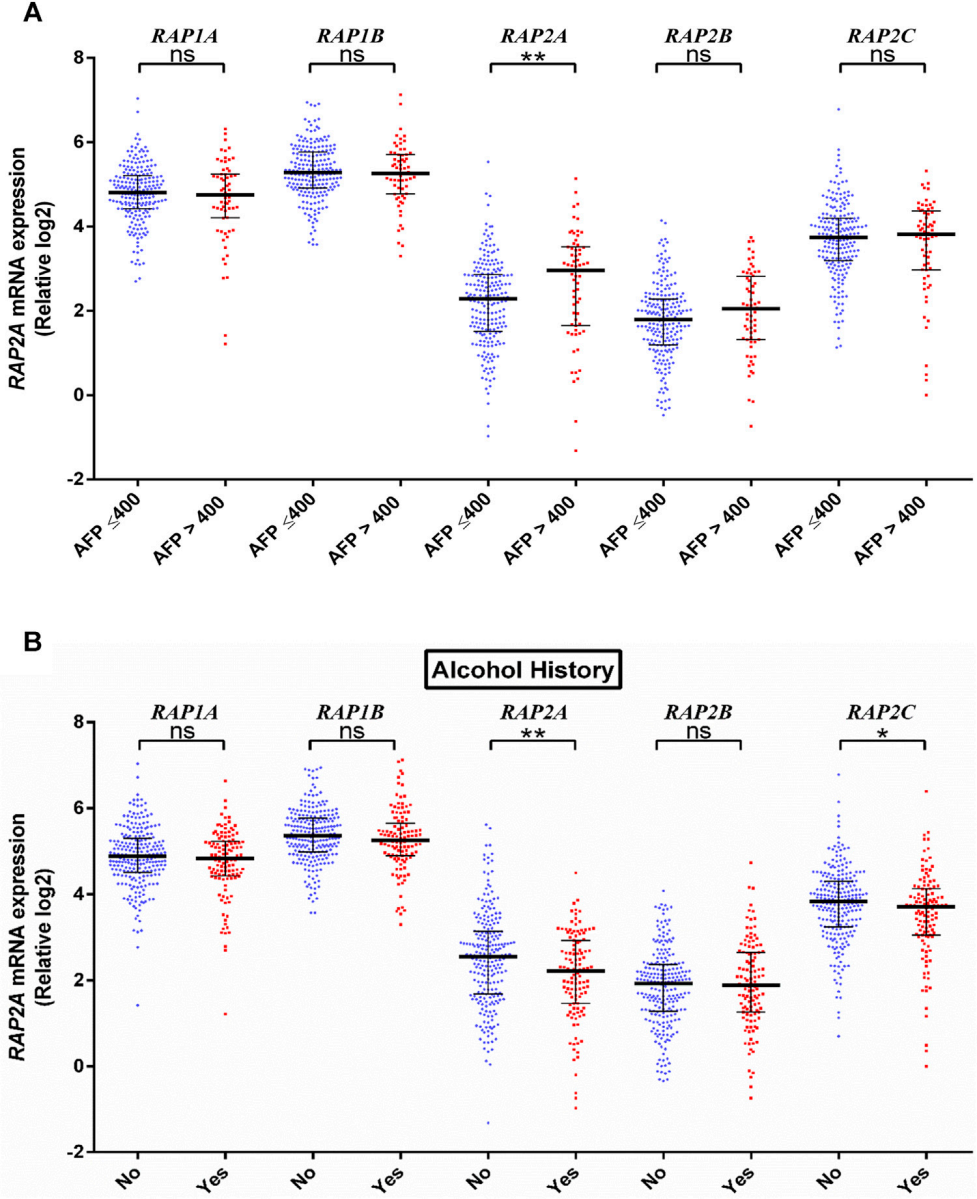
Association of RAP expression in TCGA-KIRC dataset with **(A)** AFP levels, and **(B)** alcohol history. ****p < 0.0001; ***p < 0.001; **p < 0.01; *p < 0.05; ns, *p* > 0.05.

We further assessed the association of RAP genes with clinicopathological including pathological age, gender, stage, tumor grade, blood *AFP* levels. Among all RAP genes, higher expression of RAP1B was associated with advanced-stage (Figure 3A). Higher expression of RAP2A and RAP2B, and low expression of RAP1A was associated with advanced grade (Figure 3B). High RAP2A expression was associated with younger age (<50 years, Supplementary Figure S3A) and female gender (Supplementary Figure S3B). Higher expression of RAP2A was also associated with increased AFP levels (Figure 4A). A history of alcohol consumption was associated with lower levels of RAP2A and RAP2C expression (Figure 4B).

### Association of RAP Family Expression and Genetic and Epigenetic Alterations in HCC

**FIGURE 5:**
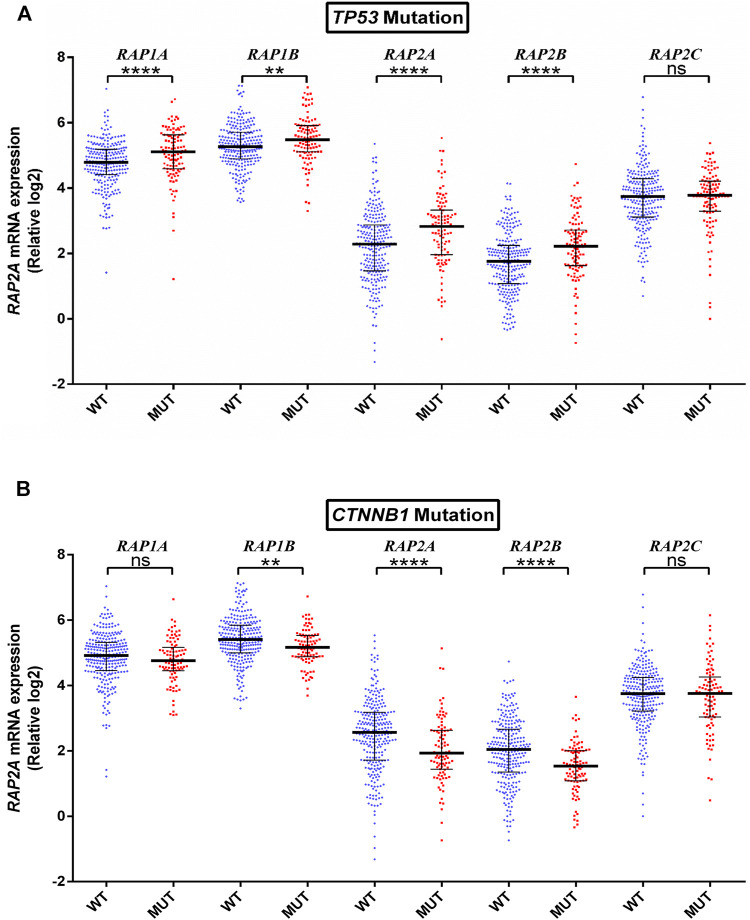
Association of RAP expression in TCGA-KIRC dataset with **(A)** TP53 mutation, and **(B**) CTNNB1 mutation. ****p < 0.0001; ***p < 0.001; **p < 0.01; *p < 0.05; ns, *p* > 0.05.

To further determine whether the expression of RAP genes is associated with genetic alterations in HCC, we compared their expression in tumors with mutated or wild type *TP53*, *CTNNB1*, *ALB*, *PCLO*, and *LRP1B*. *TP53* mutation was observed to be associated with higher expression of RAP1A, RAP1B, RAP2A, and RAP2B (Figure 5A). Further, *CTNNB1* mutation was significantly associated with reduced levels of RAP1B, RAP2A, and RAP2B expression (Figure 5B). No RAP gene exhibited association with *PCLO* and *ALB* mutation status (Supplementary Figures S4A,B respectively), while higher expression of RAP1A was associated with *LRPB1* mutant tumors (Supplementary Figure S5).

**FIGURE 6:**
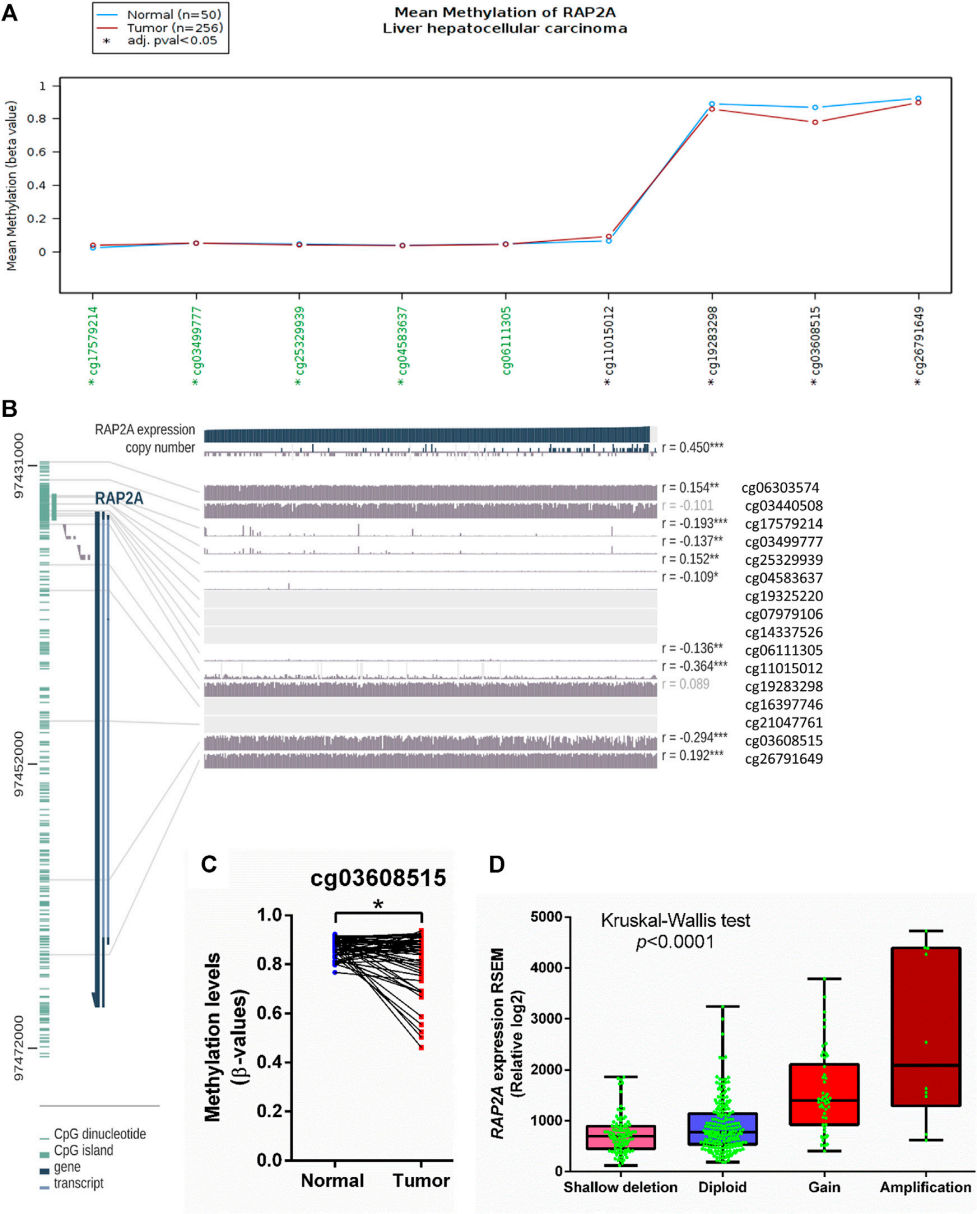
Association of mRNA expression of RAP2A with its copy number variation and DNA methylation in TCGA-LIHC dataset. **(A)** Comparison of DNA methylation level of RAP2A between tumor tissues and normal tissues. **(B)** Correlation of RAP2A mRNA expression of RAP2A with its copy number variation and DNA methylation in tumor tissues. **(C)** Comparison of DNA methylation level of RAP2A at an intragenic site associated probe cg03608515. **(D)** Comparison of RAP2A gene expression among different copy number based groups in TCGA-LIHC dataset. ***p < 0.001; **p < 0.01; *p < 0.05. Insignificant associations (*p* > 0.05), are faded.

To determine the potential role of copy number alterations and DNA methylation in the regulation of RAP2A expression in HCC, we utilized a TCGA-LIHC study where copy number variation, DNA methylation, and gene expression data were available. The DNA methylation data in TCGA study was developed on “Illumina HumanMethylation450 Beadchip” platform, where representative CpG sites from different regions of most genes are captured. Interestingly, RAP2A gene expression was reduced in tumor tissues and exhibited a negative correlation with DNA methylation at several sites within the RAP2A promoter regions and gene body (Figures 6A,B). A similar association was also observed for normal liver tissues (Supplementary Figure S6). We observed that in both normal and tumor tissues, DNA methylation at an intragenic region represented by cg03608515 was most negatively correlated with gene expression, suggesting this region, but not promoter region is the major regulatory site for the expression (Figure 6C). Furthermore, a comparison of 47 paired normal and tumor tissue also revealed significantly reduced methylation levels of cg03608515 in tumor tissues., these results strongly suggest the role of DNA methylation in aberrant expression of RAP2A in HCC. Additionally, the expression of RAP2A was also positively correlated with its copy number (r = 0.450, *p* < 0.001). Further, analysis of CNV data revealed frequent alterations in RAP2A copy number in HCC tissues was associated with its higher expression with copy number gain (Figure 6D, Kruskal-Wallis test, *p* < 0.0001).

### Prognostic Significance of RAP Genes in Hepatocellular Carcinoma

**FIGURE 7:**
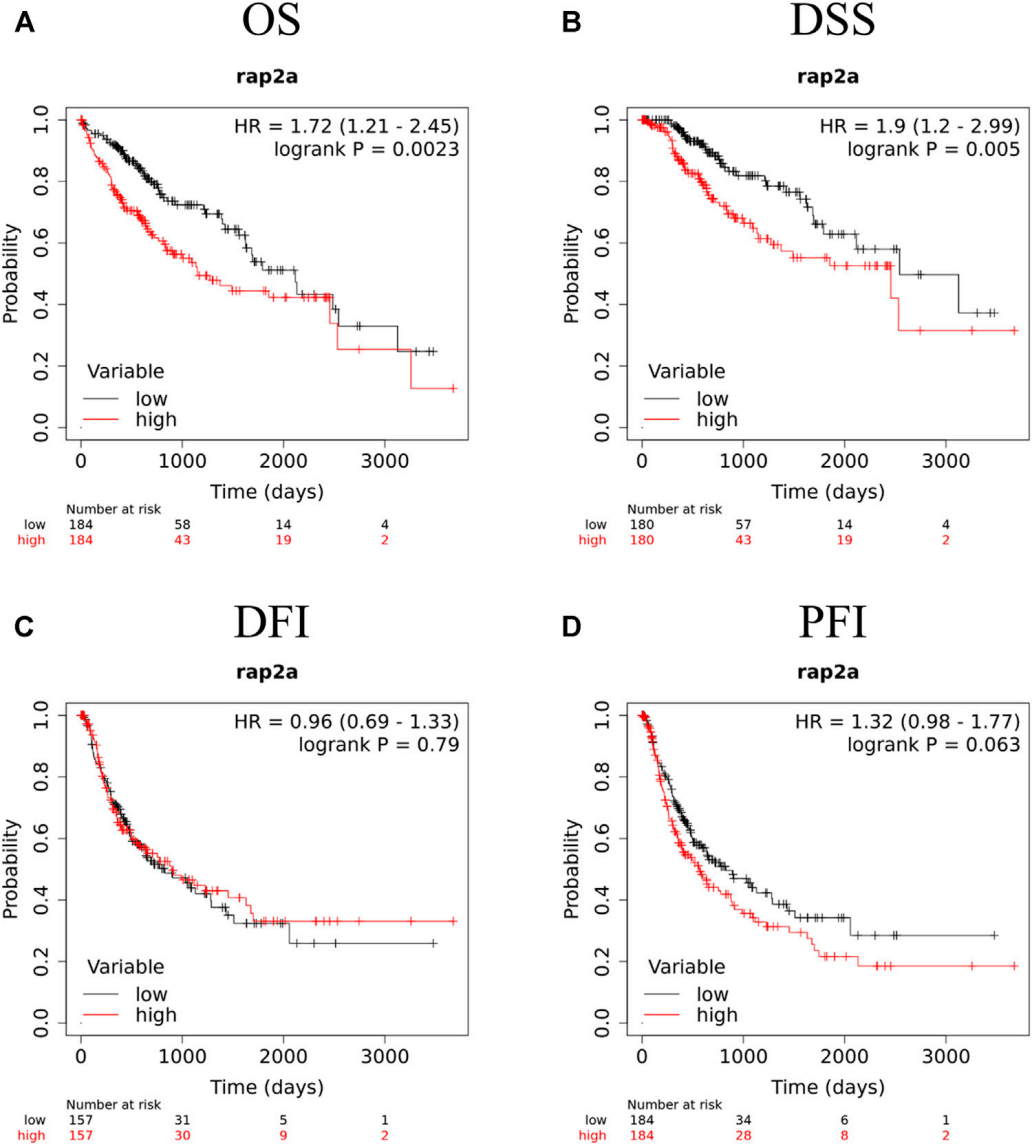
Kaplan-Meier survival analysis of RAP2A in TCGA-LIHC dataset, including **(A)** OS, overall survival **(B)** DSS, disease specific survival **(C)** DFI, disease free interval and **(D)** PFI, progression free interval. HR, hazard ratio; ****p < 0.0001; ***p < 0.001; **p < 0.01; *p < 0.05; ns, *p* > 0.05.

To determine the association of RAP gene family expression with patient prognosis, we utilized the TCGA-LIHC dataset where information for overall survival (OS), disease-specific survival (DSS), disease-free interval (DFI), and progression-free interval (PFI) was available. We performed survival analysis by constructing a Kaplan-Meier plot for all RAP genes using median expression levels for allotting patients into high and low groups. We observed that higher expression of RAP2A was significantly associated with poor OS (HR = 1.72, CI = 1.21–2.45, *p* = 0.0023, Figure 7A) and DSS (HR = 1.9, CI = 1.2–2.99, *p* = 0.005, Figure 7B), while no significant association was observed with DFI and PFI (Figures 7C,D, respectively). In light of the high positive correlation of RAP2A with other RAP genes, we also assessed their association with patient survival (Supplementary Figure S7). Among other RAPs, higher expression of RAP1A and RAP1B was also associated with poor overall survival (Supplementary Figures S7A,B). We further performed univariate and multivariate survival analysis for RAP2A expression and other clinicopathological features, such as age, gender, stage, grade, alcohol intake history, radiotherapy status, and embolization status using Cox proportionate hazard model. Interestingly, higher RAP2A expression was also associated with poor OS, DSS, and PFI in both univariate and multivariate survival analysis (Tables 2, 3, respectively). This suggested that RAP2A expression is independently associated with poor outcome in HCC patients.

**TABLE 2:** Univariate analysis for association of RAP2A expression with patient prognosis in HCC.

	OS			DSS			DFI			PFI		
	Haz. ratio	P	[95% Conf. interval]	Haz. ratio	P	[95% Conf. interval]	Haz. ratio	P	[95% Conf. interval]	Haz. ratio	P	[95% Conf. interval]
Age	1.014	0.056	1.000–1.028-	1.007	0.419	0.990–1.025	0.998	0.742	0.985–1.011	0.996	0.449	0.984–1.007
Gender	1.229	0.259	0.859–1.758	1.243	0.353	0.786–1.965	0.891	0.525	0.625–1.272	1.072	0.662	0.785–1.465
Stage 1	(Ref.)			(Ref.)			(Ref.)			(Ref.)		
2	1.535	0.086	0.941–2.504	1.734	0.118	0.869–3.462	1.708	0.014	1.116–2.614	1.943	0.001	1.321–2.857
3	2.728	0.000	1.774–4.193	4.169	0.000	2.342–7.424	2.829	0.000	1.876–4.265	2.721	0.000	1.874–3.952
4	5.318	0.002	1.892–14.950	9.331	0.000	2.731–31.878	23.214	0.002	3.055–176.362	6.951	0.000	2.483–19.456
Grade 1	(Ref.)			(Ref.)			(Ref.)			(Ref.)		
2	1.269	0.387	0.740–2.175	1.316	0.443	0.653–2.653	1.489	0.156	0.859–2.582	1.189	0.451	0.758–1.865
3	1.268	0.409	0.721–2.230	1.413	0.351	0.683–2.924	1.724	0.056	0.986–3.015	1.347	0.209	0.846–2.142
4	1.514	0.458	0.507–4.519	0.689	0.724	0.088–5.411	1.002	0.998	0.291–3.446	0.920	0.877	0.320–2.647
Embolization	0.859	0.633	0.461–1.602	1.350	0.361	0.709–2.568	2.302	0.000	1.443–3.674	2.218	0.000	1.457– 3.375
Radiation	0.959	0.943	0.304–3.021	0.986	0.984	0.241–4.024	1.590	0.310	0.649–3.892	1.544	0.297	0.683–3.494
Alcohol history	1.050	0.799	0.719–1.535	1.466	0.099	0.930–2.311	1.130	0.502	0.791–1.616	1.043	0.794	0.760–1.432
RAP2A	**1.325**	**0.000**	1.132–1.550	**1.429**	**0.001**	1.166–1.750	1.099	0.216	0.946–1.276	**1.189**	**0.011**	1.040–1.359

OS, overall survival; DSS, disease-specific survival; DFI, disease-free interval; PFI, progression-free interval; HR, hazard ratio; CI, confidence interval.

**TABLE 3:** Multivariate analysis for association of RAP2A expression with patient prognosis in HCC.

	OS			DSS			DFI			PFI		
	Haz. ratio	P	[95% Conf. interval]	Haz. ratio	P	[95% Conf. interval]	Haz. ratio	P	[95% Conf. interval]	Haz. ratio	P	[95% Conf. interval]
Age	1.028	0.003	1.009–1.047	1.009	0.410	0.987–1.032	1.002	0.809	0.986–1.018	1.000	0.982	0.987–1.013
Gender	0.971	0.899	0.611–1.542	1.136	0.679	0.622–2.073	0.873	0.540	0.564–1.349	0.980	0.918	0.662–1.449
Stage 1	(Ref.)			(Ref.)			(Ref.)			(Ref.)		
2	1.615	0.096	0.918–2.839	2.143	0.047	1.012–4.538	1.895	0.010	1.165–3.082	2.100	0.001	1.358–3.246
3	2.851	0.000	1.775–4.581	4.439	0.000	2.405–8.193	3.901	0.000	2.451–6.210	3.311	0.000	2.193–5.000
4	5.230	0.030	1.176–23.267	7.137	0.012	1.532–33.252	33.053	0.001	4.123–265.000	8.346	0.001	2.362–29.483
Grade 1	(Ref.)			(Ref.)			(Ref.)			(Ref.)		
2	1.126	0.726	0.580–2.185	1.849	0.191	0.736–4.648	1.505	0.211	0.793–2.855	1.173	0.568	0.678–2.028
3	1.323	0.418	0.672–2.603	1.837	0.209	0.712–4.741	1.837	0.067	0.957–3.527	1.224	0.484	0.695–2.154
4	1.593	0.487	0.429–5.924	1.479	0.725	0.168–13.052	0.979	0.978	0.214–4.487	0.886	0.850	0.253–3.101
Embolization	1.017	0.966	0.470–2.203	1.661	0.224	0.733–3.766	3.658	0.000	2.075–6.446	2.966	0.000	1.746–5.037
Radiation	1.057	0.928	0.319–3.498	1.031	0.967	0.239–4.448	0.996	0.993	0.388–2.556	1.185	0.698	0.503–2.789
Alcohol history	0.955	0.849	0.597–1.529	1.682	0.085	0.931–3.040	1.077	0.740	0.696–1.667	1.244	0.270	0.844–1.834
RAP2A	**1.296**	**0.011**	1.062–1.581	**1.334**	**0.028**	1.032–1.724	1.063	0.528	0.879–1.287	**1.199**	**0.037**	1.011–1.423

OS, overall survival; DSS, disease-specific survival; DFI, disease-free interval; PFI, progression-free interval; HR, hazard ratio; CI, confidence interval.

### RAP2A Associated Cellular Pathways in Hepatocellular Carcinoma

**FIGURE 8:**
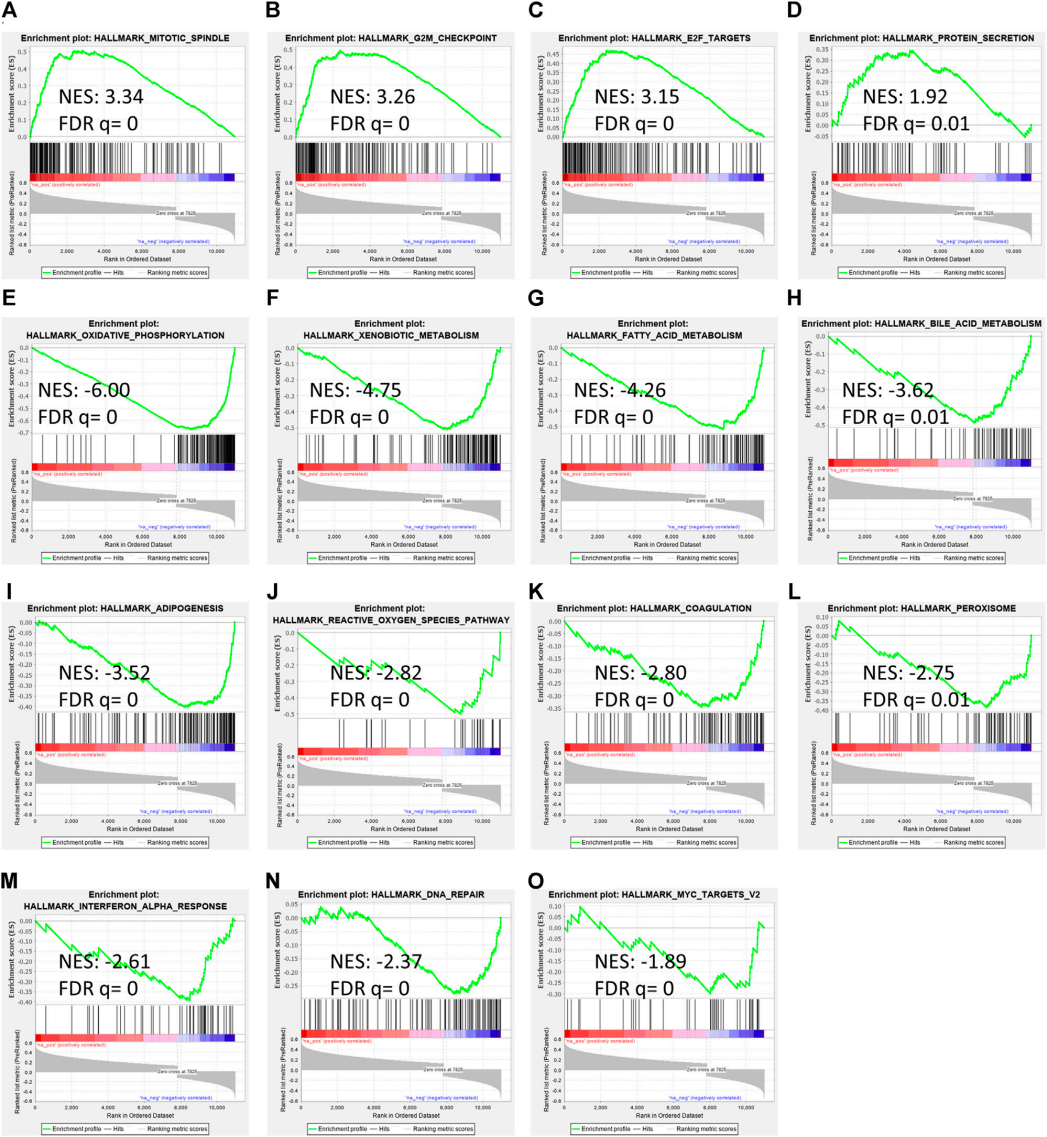
Gene set enrichment analysis of RAP2A correlated genes in TCGA-LIHC dataset. **(A–D)** depicts positively enriched pathways **(E–L)** depicts negatively enriched pathways with normalized enrichment score (NES), false discovery rate (FDR), and *p*-value depicted inside the respective pathway.

To determine RAP2A associated cancer-related pathways, gene expression data of the TCGA-LIHC study was used. GSEA analysis revealed that RAP2A expression is positively correlated with cell cycle associated pathways such as mitotic spindle (Figure 8A), G2M checkpoint (Figure 8B), and E2F targets (Figure 8C) besides protein secretion (Figure 8D). Further, negatively correlated genes were enriched in metabolism associated pathways, such as oxidative phosphorylation (Figure 8E), xenobiotic metabolism (Figure 8F), fatty acid metabolism (Figure 8G), bile acid metabolism (Figure 8H), adipogenesis (Figure 8I), reactive oxygen species (Figure 8J) and others such as coagulation (Figure 8K), peroxisome (Figure 8L), interferon-alpha response (Figure 8M), DNA repair (Figure 8N) and Myc target genes (Figure 8O).

### Association of RAP2A Expression With Tumor Immunity

**FIGURE 9:**
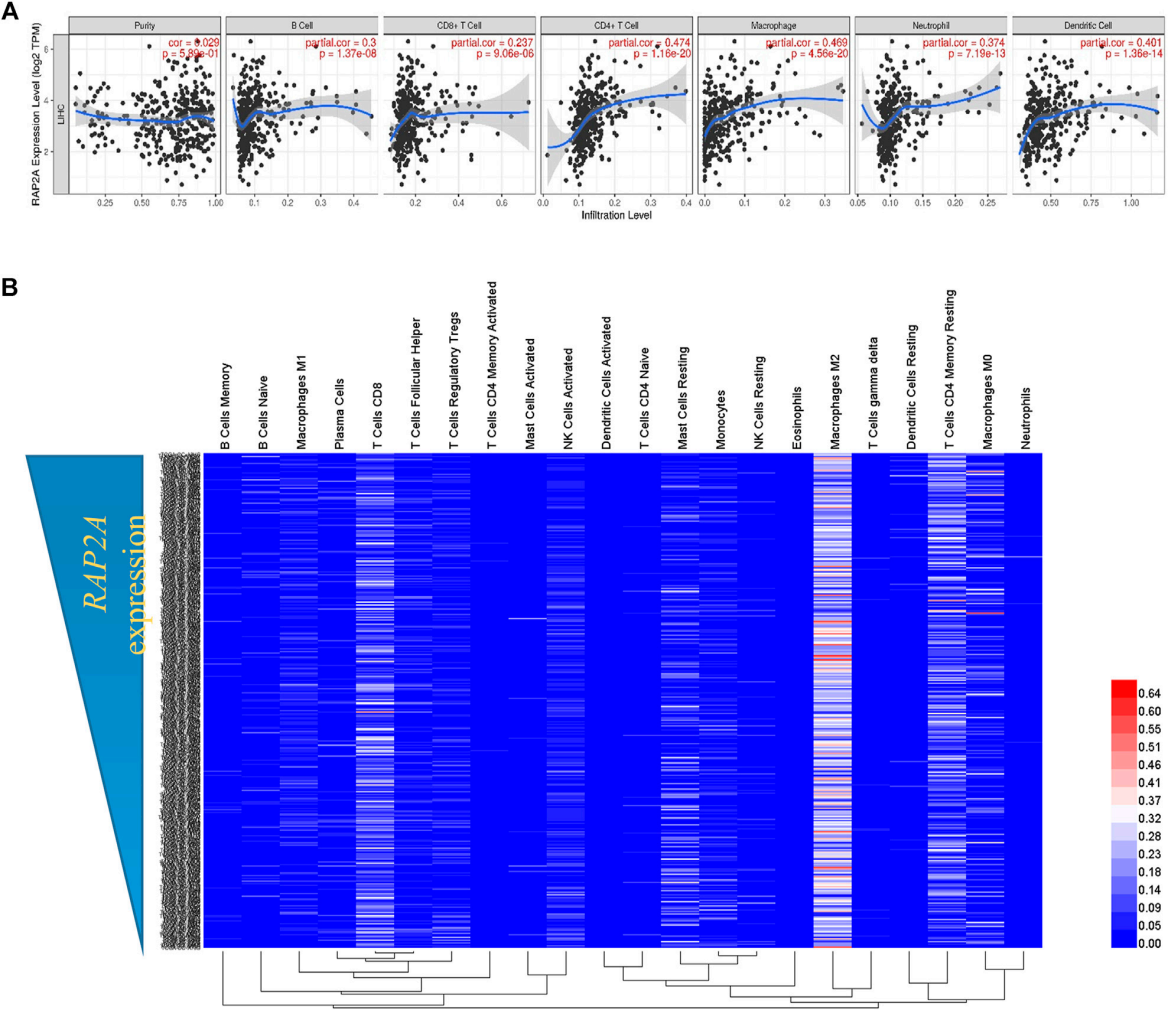
Association of RAP2A gene with tumor immunity in TCGA-LIHC dataset. **(A)** TIMER analysis showing the correlation of RAP2A expression with an abundance of six different immune cell types in TCGA-LIHC dataset. **(B)** CIBERSORT analysis showing relative fractions of 22 different immune cell types in HCC tissues (represented by rows) arranged in order of high RAP2A expression (top) to low RAP2A expression (bottom).

Considering the previously described role of RAP genes in immune cell functions (Carvalho et al., 2019a), we analyzed the association of RAP2A expression with the level of immune cell infiltration. Using the TIMER tool, we determine tumor purity normalized spearman correlation of RAP2A expression with infiltration level of six different immune cells. This analysis revealed a positive correlation between RAP2A expression with B cells (r = 0.3, *p* = 1.37e-08), CD8+ T cells (r = 0.237, *p* = 9.06e-06), CD4+ T cells (r = 0.474, *p* = 1.16e-20), macrophages (r = 0.469, *p* = 4.56e-20), neutrophils (r = 0.374, *p* = 7.19e-13), and dendritic cells (r = 0.401, *p* = 1.36e-14) in HCC (Figure 9A). Furthermore, we utilized CIBERSORT analysis to determine the association of RAP2A gene expression with the relative abundance of 22 different types of immune cells in the TCGA-LIHC dataset (Figure 9B, Supplementary Table S2). Among immune cells, RAP2A expression was positively correlated to CD4 Memory Resting T cells, resting dendritic cells, neutrophils, M0 type macrophages, and naïve B cells, while it exhibited negative correlations to monocytes, activated NK cells, CD4 naïve T cells, CD8 T cells.

## Discussion

HCC is one of the leading causes of cancer-related deaths worldwide. Significant advancement has been made in the treatment of this malignancy over the past decade, however, clinical response is highly heterogeneous. Further, treatment strategies have been highly adapted to be based on the progression of the disease at the time of diagnosis. Nevertheless, several molecular biomarkers have been determined with high prognostic value and future studies are required to determine novel molecular features as therapeutic targets and prognostic biomarkers. In the current study, we uncovered distinct genomic and epigenomic features of RAP family genes in HCC. Our study revealed that among five RAP genes, RAP2A expression is highly altered in HCC and is associated with multiple oncogenic features in HCC.

Little is known about the specific roles of RAP2A; in its active form RAP2A interacts with several effectors including MINK1, TNIK, and MAP4K4 and activates various signaling pathways involved in cytoskeletal rearrangements, cell migration, cell adhesion, and cell proliferation (Mittal and Linder, 2006). RAP2A interacts directly with upstream MAPK signaling element MAP4K4, and thus, increased RAP2A activity can enable downstream signaling (Machida et al., 2004). So far, the role of RAP2A in human malignancies remains controversial, with some suggesting it as a tumor suppressor gene while other studies refer to it as an oncogene. Upregulation of RAP2A has been observed in several human malignancies such as follicular thyroid cancer (Prabakaran et al., 2011), prostate cancer (Bigler et al., 2007), renal cancer (Wu et al., 2017), gastric cancer (Zhang J. et al., 2020) and bladder cancer (Wang et al., 2020).

In prostate cancer cells, RAP2A promotes androgen hypersensitivity and cell growth (Bigler et al., 2007). In lung cancer cells, ectopic expression of RAP2A enhances the migration and invasion of the cells (Wu et al., 2014). In bladder cancer cells, the expression of RAP2a was found significantly higher as compared to normal cells. The proliferation and invasion of cells were repressed by miR-3127 through directly targeting the 3′-UTR of RAP2A and associated with poor overall survival in bladder cancer patients (Wang et al., 2020). In gastric cancer, the role of RAP2A was also observed in drug resistance where expression of RAP2A increased the viability, migration, and metastasis of cells by suppressing apoptosis and DNA damage (Zhang J. et al., 2020). In renal cancer, overexpression of RAP2A enhances the protein levels of p-Akt and promotes migration and invasion of cells by increasing p-Akt expression (Wu et al., 2017). Contrary to these, RAP2A seems to play tumor suppressor functions in glioma as its downregulation is associated with glioma progression and its inhibition in the glioma cell line reduces migration and invasion (Wang et al., 2014). Results of the current study indicate that in hepatocellular carcinoma, RAP2A may act as an important oncogene and its mRNA expression is strongly associated with patient prognosis in HCC. Furthermore, other RAP genes also exhibit a strong positive correlation with RAP2A expression. This might be due to the conservation of regulatory sequences during evolution. We were further interested in whether RAP genes share common features for association with molecular characteristics in HCC.

It was recently demonstrated that RAP2A expression is regulated by p53 and RAP2A mediated cell migration and invasive properties are driven by downstream activation of the matrix metalloproteinases (MMP) MMP2 and MMP9 via phosphorylation of AKT (Wu et al., 2015). Consistent with this, we observed higher expression of multiple RAP genes, including RAP2A in p53 mutant HCC. Further, we also observed that expression of RAP1A, RAP1B, RAP2A, and RAP2B were reduced in HCC tissues which harbor a mutation in *CTNNB1*, the gene encoding for beta-catenin protein. This is contrary with the previous report where RAP1B has shown to activate Wnt/beta-catenin signaling in esophageal squamous cell carcinoma (Jia et al., 2017). Further, RAPGEF2, a guanine nucleotide exchange factor for RAP1, was shown to regulate adherence junction (AJ) formation in radial glial cells through ERK-mediated upregulation of β-catenin (Farag et al., 2017). While *CTNNB1* mutations in HCC are associated with higher activity of Wnt-beta catenin signaling (Tornesello et al., 2013), its association with RAP signaling appears to be negatively related in this case. Therefore, our results suggested potential crosstalk of Wnt-beta catenin signaling in RAP signaling in HCC tissues.

In light of its aberrant overexpression in HCC, we explored whether the expression of RAP2A is driven by copy number alteration and DNA methylation in HCC. Our results collectively demonstrated that the RAP2A harbors alterations in both of the abovementioned features. Our results highlighted a specific intragenic region in the RAP2A where DNA methylation was highly reduced in tumor tissues compared to normal liver tissues. Further, DNA methylation at this region is negatively correlated to RAP2A gene expression in both tumor and normal tissues. DNA methylation of RAP2A has not been previously studied in cancer, therefore, epigenetic regulation of RAP signaling requires detailed exploration.

While our study is based on mRNA expression, a recent study by, Zheng et al. has also demonstrated that RAP2A protein expression is associated with oncogenic features in HCC (Zheng et al., 2017). Therefore, our findings further provide a detailed understanding of the role of all five members of this gene family involvement in HCC. Among all five RAPs, RAP2A expression exhibited a strong ability to differentiate tumor tissues from normal tissues. Further, its higher expression also exhibited association with higher tumor grade, metastasis, increased AFP levels, and poor patient prognosis. Furthermore, our multivariate survival analysis including major clinical and pathological features revealed that the RAP2A expression is independently associated with poor overall survival, disease-specific survival, and progression-free interval in HCC.

Pathway analysis revealed strong associations of RAP2A expression in HCC with several HCC relevant pathways, including cell cycle-related pathways and metabolic pathways. Interestingly, RAP1A expression has previously been shown to be regulated during the cell cycle (Cruise et al., 1997). The causal relationship between RAP2A expression and these pathways requires further validation. We also analyzed the immunological association of RAP2A expression in HCC, which revealed that its expression is highly associated with the immune composition of HCC tumors. While, the role of RAP2A has been previously demonstrated in the regulation of lipopolysaccharide induced innate cell functions (Carvalho et al., 2019a; Carvalho et al., 2019b), detailed role of RAP2A in the modulation of tumor immunity remains to be studied in detail. Conclusively, the current study provides detailed molecular and clinical features associated with the expression of RAP genes in HCC, however, some of these associations require further exploration for the causal relationships. Further, these results support the potential of RAP2A as a therapeutic target and prognostic biomarker in this malignancy.

## Data Availability

The datasets presented in this study can be found in online repositories. The names of the repository/repositories and accession number(s) can be found in the article/Supplementary Material.
