# Supplementary material for: Molecular Associations and Clinical Significance of RAPs in Hepatocellular Carcinoma
Source: Front Mol Biosci. 2021 Jun 21;8:677979. doi: 10.3389/fmolb.2021.677979 (PMC8255377; doi:10.3389/fmolb.2021.677979)
Supplement: Supplementary file 1 [file DataSheet1.docx]

**Supplementary Table 1: Correlation matrix of expression of RAP family genes in TCGA-LIHC dataset.**

|  | *RAP1A* | *RAP1B* | *RAP2A* | *RAP2B* | *RAP2C* |
| --- | --- | --- | --- | --- | --- |
| *RAP1A* | 1 | 0.6880255 | 0.5820059 | 0.5664384 | 0.468875 |
| *RAP1B* | 0.6880255 | 1 | 0.6239759 | 0.6321911 | 0.5022693 |
| *RAP2A* | 0.5820059 | 0.6239759 | 1 | 0.568841 | 0.4978313 |
| *RAP2B* | 0.5664384 | 0.6321911 | 0.568841 | 1 | 0.3602865 |
| *RAP2C* | 0.468875 | 0.5022693 | 0.4978313 | 0.3602865 | 1 |

**Supplementary Table 2: Correlation of expression of RAP2A with immune cell fractions estimated by CIBERSORT analysis.**

| **Cell type** | **r** | **95% confidence interval** | **P (two-tailed)** | **P value summary** |
| --- | --- | --- | --- | --- |
| Monocytes | -0.1608 | -0.2627 to -0.05535 | 0.0022 | ** |
| NK Cells Activated | -0.1467 | -0.2492 to -0.04091 | 0.0053 | ** |
| T Cells CD4 Naive | -0.1369 | -0.2398 to -0.03099 | 0.0093 | ** |
| T Cells CD8 | -0.1204 | -0.2240 to -0.01421 | 0.0223 | * |
| Mast Cells Resting | -0.099 | -0.2032 to 0.007478 | 0.0606 | ns |
| NK Cells Resting | -0.08423 | -0.1889 to 0.02236 | 0.1106 | ns |
| T Cells gamma delta | -0.07247 | -0.1775 to 0.03419 | 0.1701 | ns |
| Macrophages M2 | -0.03746 | -0.1433 to 0.06922 | 0.4787 | ns |
| Mast Cells Activated | -0.0374 | -0.1432 to 0.06927 | 0.4794 | ns |
| Dendritic Cells Activated | -0.03154 | -0.1375 to 0.07511 | 0.5509 | ns |
| B Cells Memory | -0.01552 | -0.1217 to 0.09103 | 0.7692 | ns |
| T Cells Regulatory Tregs | 0.00814 | -0.09834 to 0.1144 | 0.8777 | ns |
| Eosinophils | 0.008545 | -0.09794 to 0.1148 | 0.8716 | ns |
| T Cells CD4 Memory Activated | 0.01375 | -0.09278 to 0.1200 | 0.7948 | ns |
| T Cells Follicular Helper | 0.03393 | -0.07273 to 0.1398 | 0.521 | ns |
| Plasma Cells | 0.04174 | -0.06494 to 0.1475 | 0.4298 | ns |
| Macrophages M1 | 0.08555 | -0.02104 to 0.1902 | 0.1051 | ns |
| B Cells Naive | 0.1257 | 0.01961 to 0.2291 | 0.017 | * |
| Macrophages M0 | 0.1329 | 0.02684 to 0.2359 | 0.0116 | * |
| Neutrophils | 0.1404 | 0.03455 to 0.2432 | 0.0076 | ** |
| Dendritic Cells Resting | 0.1448 | 0.03903 to 0.2474 | 0.0059 | ** |
| T Cells CD4 Memory Resting | 0.1945 | 0.08994 to 0.2948 | 0.0002 | *** |


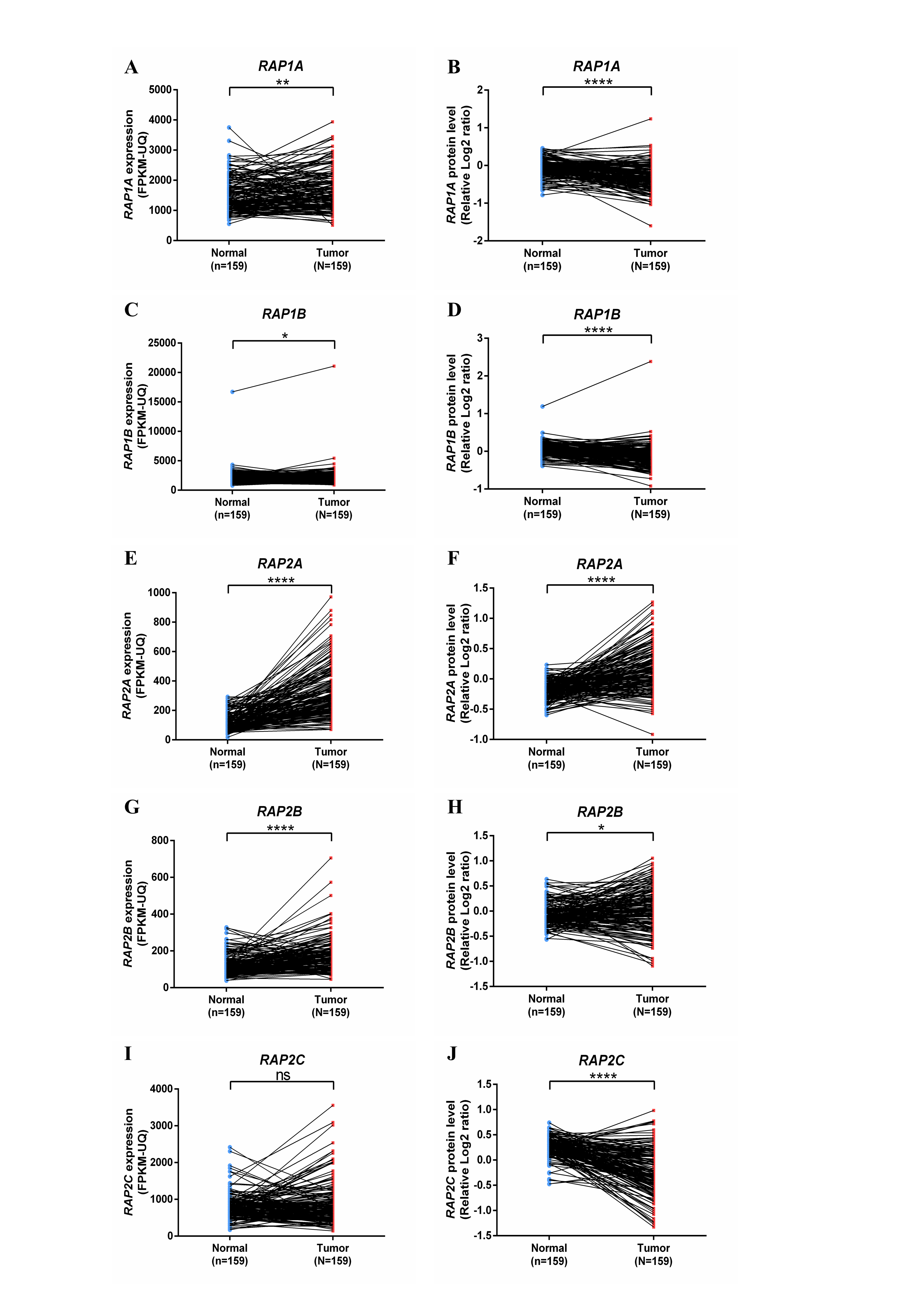


**Supplementary Figure S1:** Expression pattern of RAP gene family in hepatocellular carcinoma tissues compared to normal tissues CPTAC liver cancer dataset. Left panel, mRNA levels; Right panel, protein levels. (A-B) RAP1A (C-D) RAP1B (E-F) RAP2A (G-H) RAP2B (I-J) RAP2C.


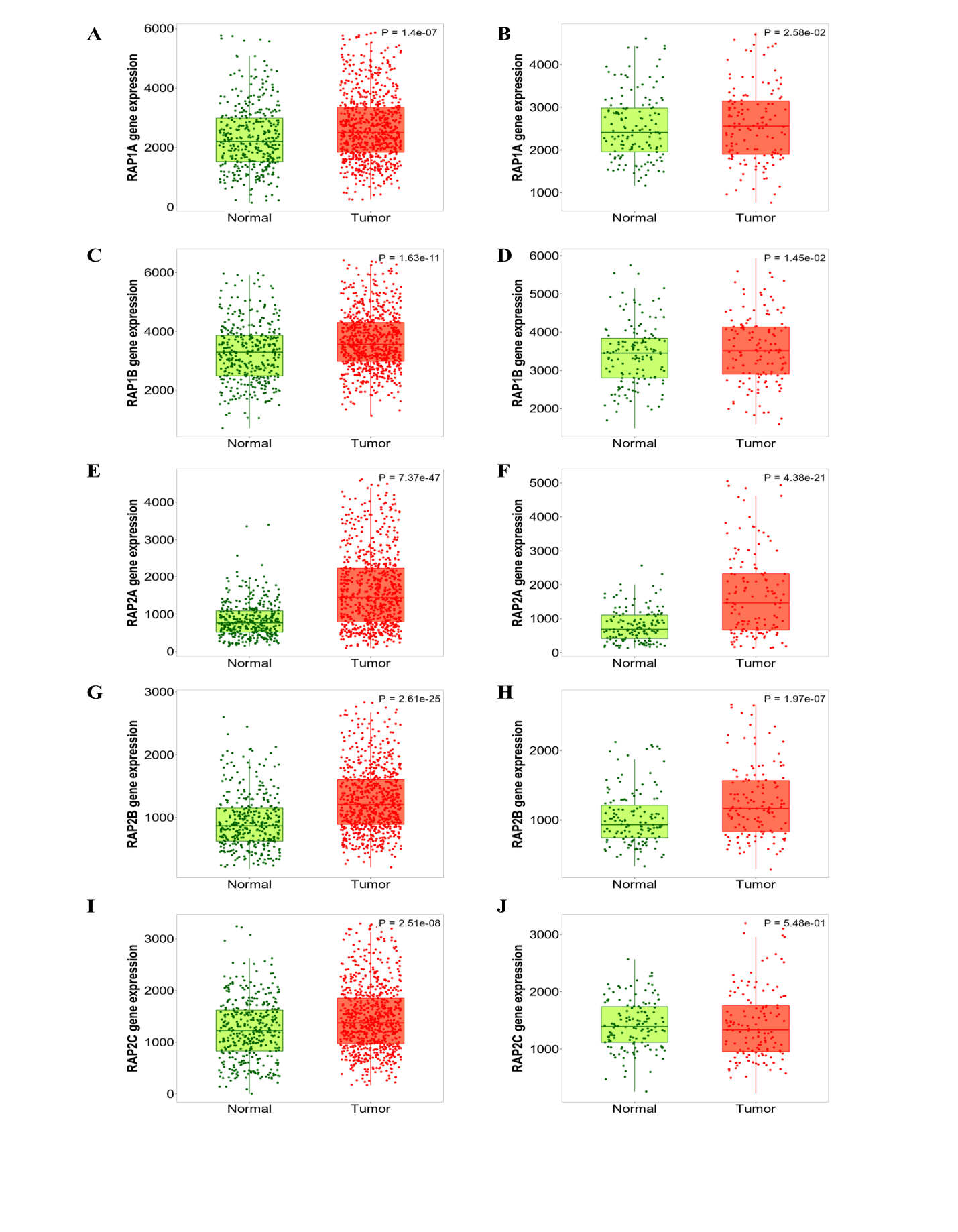


**Supplementary figure S2:** Expression pattern of RAP gene family in hepatocellular carcinoma tissues compared to normal tissues. Left panel, unpaired samples; Right panel, paired sample analysis. (A-B) RAP1A (C-D) RAP1B (E-F) RAP2A (G-H) RAP2B (I-J) RAP2C.


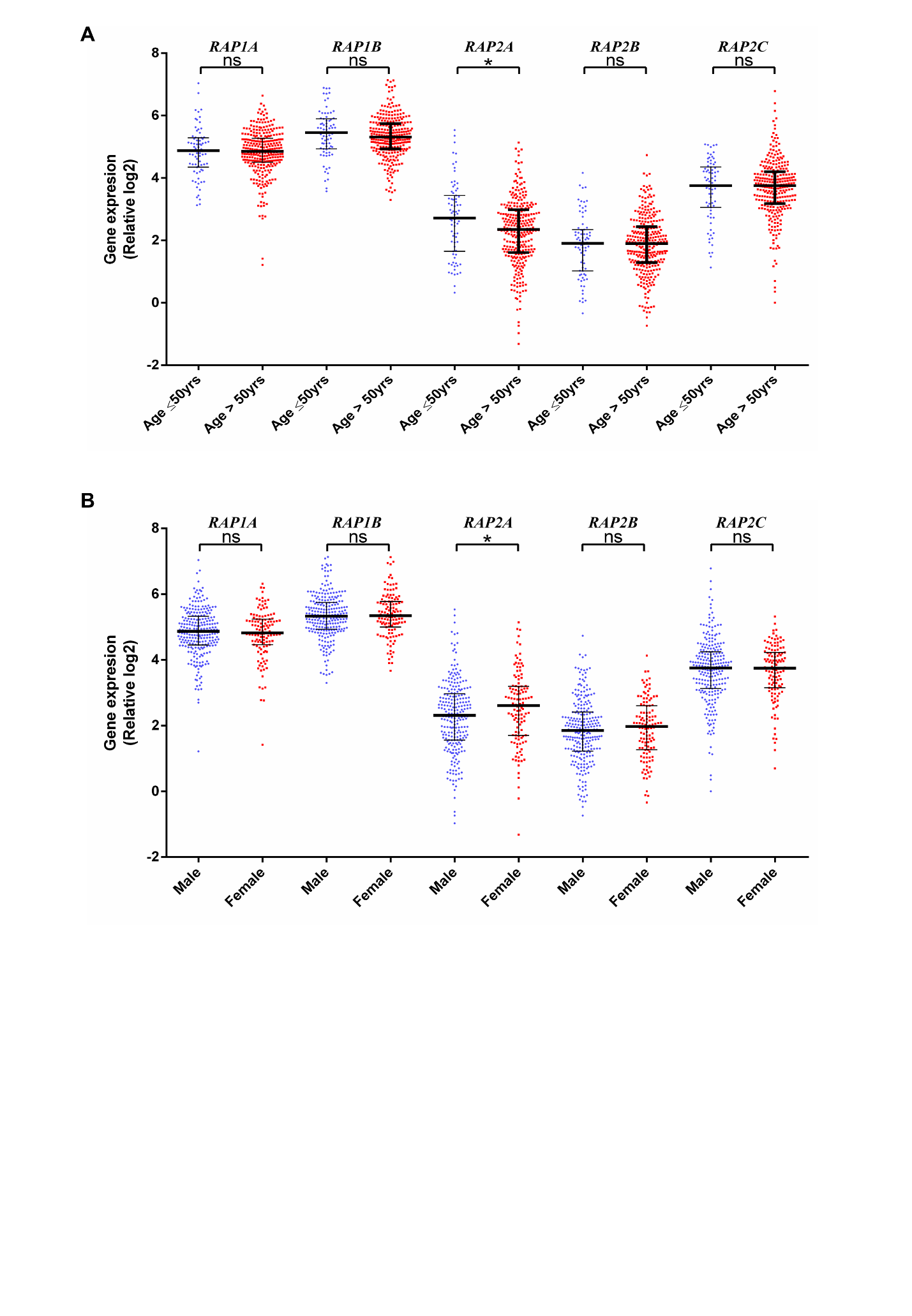


**Supplementary Figure S3:** Association of RAP gene family expression in TCGA-LIHC dataset with (A) Age and (B) Gender. ****p<0.0001; ***p<0.001; **p<0.01; *p<0.05; ns, p>0.05.


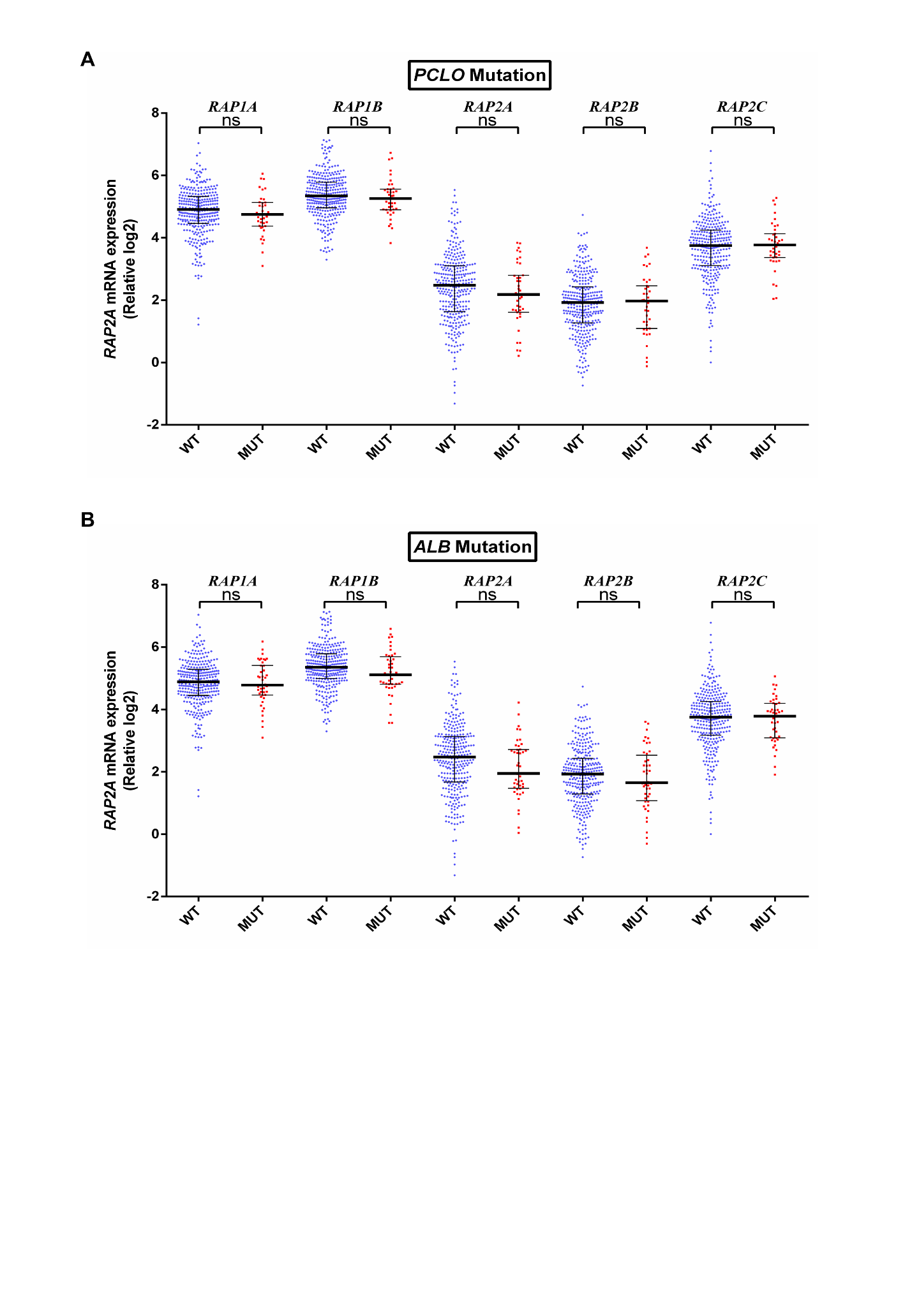


**Supplementary Figure S4:** Association of RAP gene family expression in TCGA-LIHC dataset with (A) PCLO mutation and (B) ALB mutation. ****p<0.0001; ***p<0.001; **p<0.01; *p<0.05; ns, p>0.05.


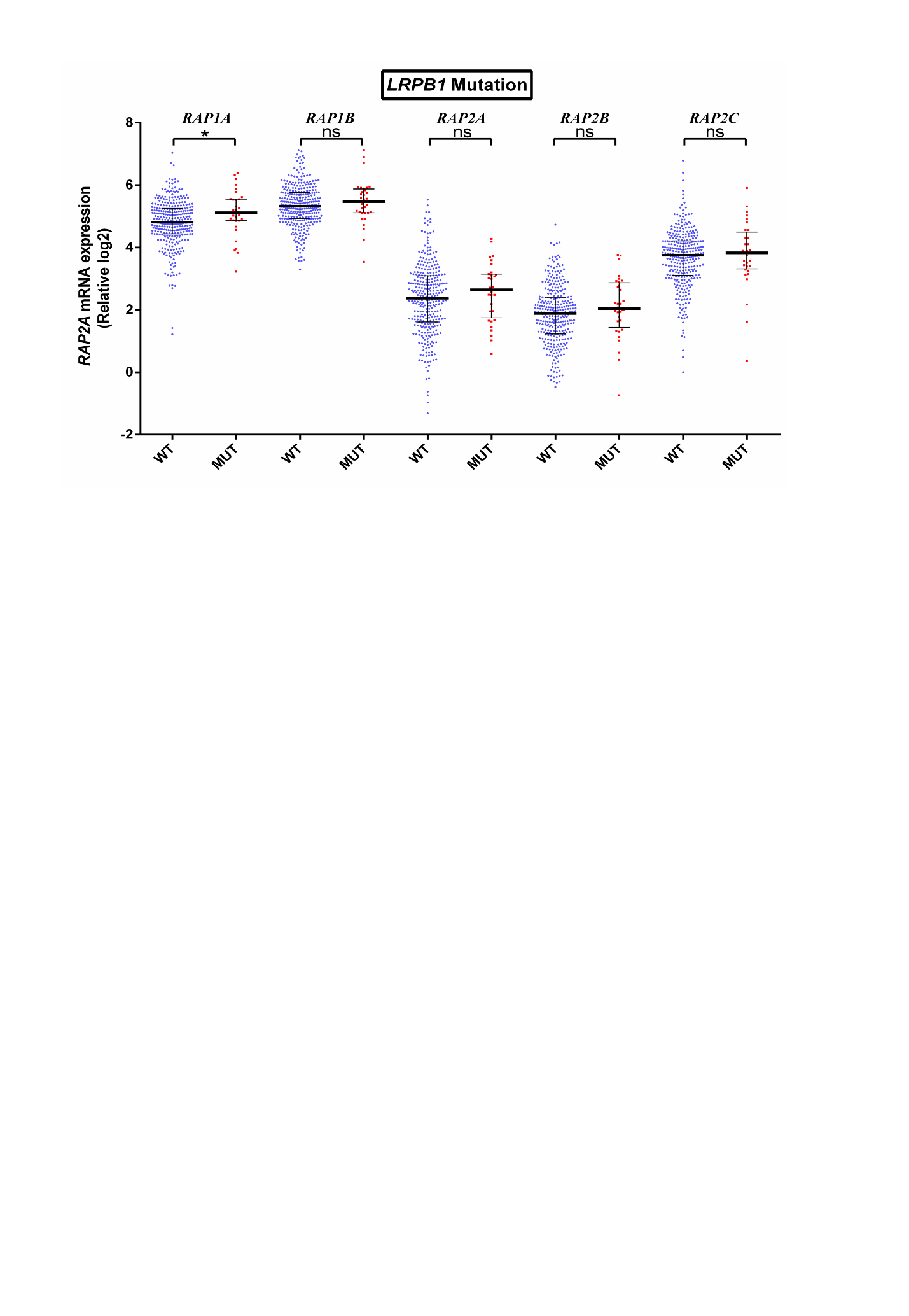


**Supplementary Figure S5:** Association of RAP gene family expression in TCGA-LIHC dataset with *LRPB1* mutation. *p<0.05; ns, p>0.05.


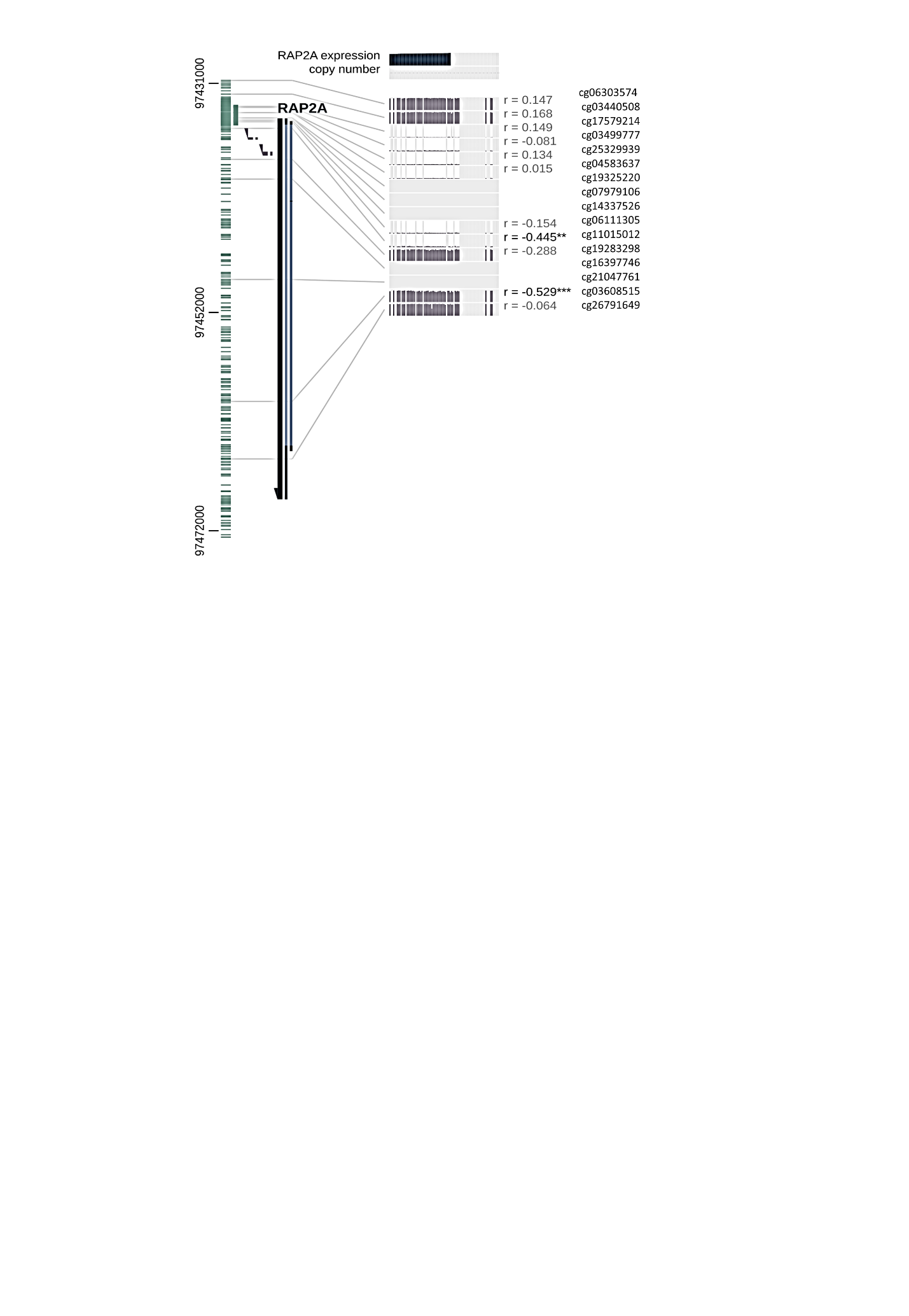


**Supplementary Figure S6:** Correlation of RAP2A mRNA expression of RAP2A with DNA methylation in normal tissues. ***p<0.001; **p<0.01; *p<0.05. Insignificant associations (p>0.05), are faded.


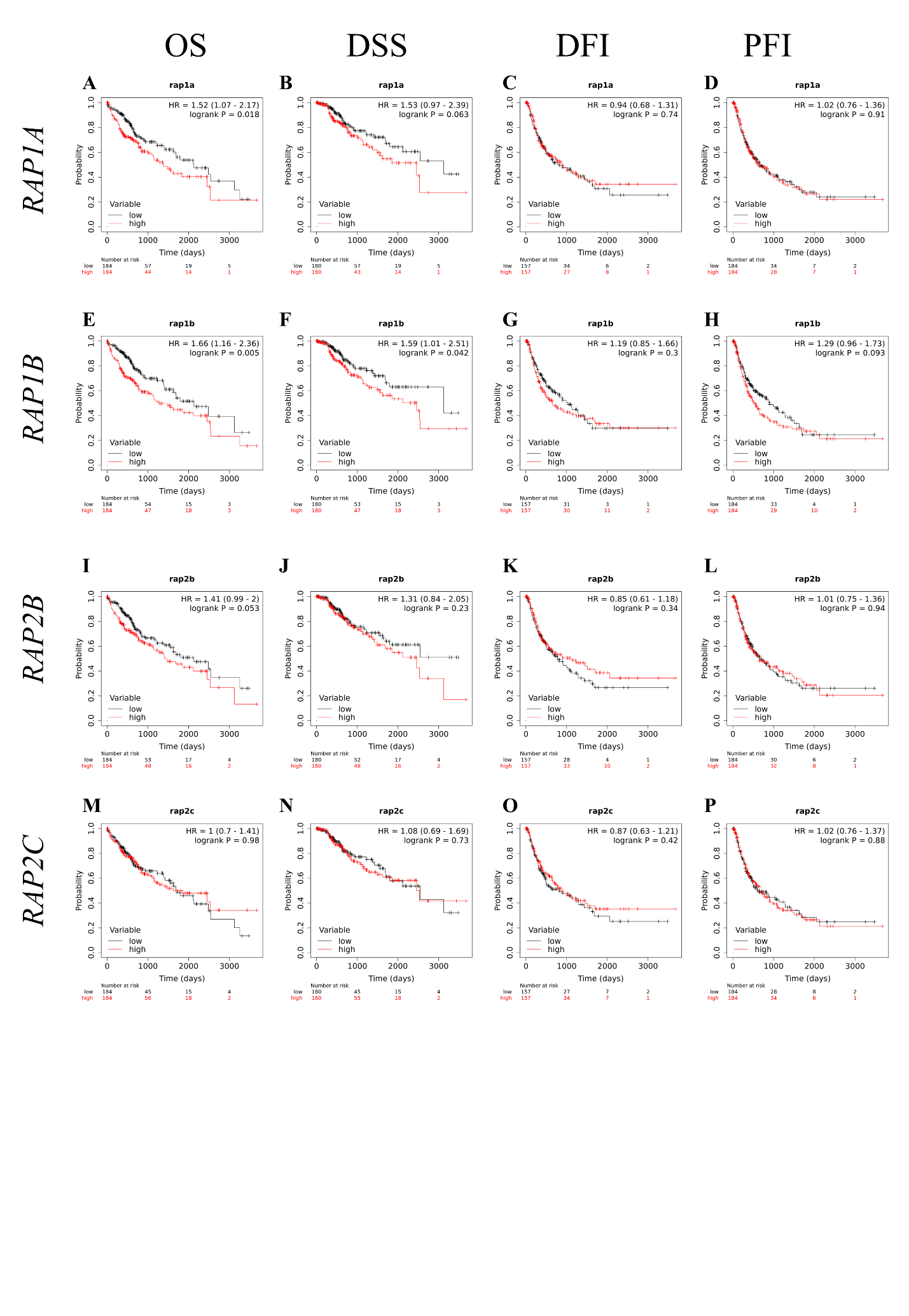


**Supplementary Figure S7:** Kaplan-Meier survival analysis of RAP1A, RAP1B, RAP2B and RAP2C in TCGA-LIHC dataset, including overall survival (OS), disease specific survival (DSS), disease free interval and (DFI), and progression free interval (PFI). HR, hazard ratio; ****p<0.0001; ***p<0.001; **p<0.01; *p<0.05; ns, p>0.05.
